# Identification of SEC61G as a Diagnostic and Prognostic Biomarker in Oral Squamous Cell Carcinoma

**DOI:** 10.3390/biomedicines11102718

**Published:** 2023-10-06

**Authors:** Shi-Long Zhang, Lei Chen, Lin-Lin Bu, Zi-Li Yu, Si-Rui Ma

**Affiliations:** 1 State Key Laboratory of Oral & Maxillofacial Reconstruction and Regeneration, Key Laboratory of Oral Biomedicine Ministry of Education, Hubei Key Laboratory of Stomatology, School & Hospital of Stomatology, Wuhan University, Wuhan 430079, China; zhangshilong92@hotmail.com (S.-L.Z.); chenlei199441@163.com (L.C.); lin-lin.bu@whu.edu.cn (L.-L.B.); 2 Department of Oral and Maxillofacial Surgery, Dongfeng Stomatological Hospital, Hubei University of Medicine, Shiyan 442000, China; 3 Department of Oral and Maxillofacial-Head and Neck Oncology, School and Hospital of Stomatology, Wuhan University, Wuhan 430079, China

**Keywords:** oral squamous cell carcinoma, SEC61G, prognosis, immune infiltration

## Abstract

Oral squamous cell carcinoma (OSCC) is a heterogeneous malignancy originating from the oral mucosal epithelium. Detecting novel biomarkers can offer crucial information on disease aggressiveness and expected clinical outcomes for individual patients. SEC61G, an aberrantly expressed gene in various cancers, has been associated with negative clinical outcomes. However, its expression and clinical significance in OSCC is still unclear. In the present study, we investigated the SEC61G expression level in OSCC using bioinformatic and immunohistochemical analyses. Additionally, our findings revealed a significant correlation between SEC61G expression and clinicopathological characteristics, as well as a worse prognosis in OSCC patients. Notably, flow cytometry analysis on patient samples revealed that SEC61G expression was also linked to decreased immune infiltration in OSCC patients. In conclusion, our study provides evidence supporting SEC61G’s role as a potential diagnostic, prognostic, and therapeutic marker in OSCC.

## 1. Introduction

Oral squamous cell carcinoma (OSCC) is a subgroup of malignancies that originate from the mucosal epithelium of the oral cavity. It is a heterogeneous malignancy that can vary in clinical presentation and behavior [1,2]. OSCC is typically treated based on the stage of the disease. The primary treatment approach is surgical resection. In more advanced cases, radiation or radiation therapy combined with chemotherapy, known as chemoradiation (CRT), may be recommended [3]. Although there have been advancements in treatment options, such as target therapy and immunotherapy, for OSCC over the past few decades, these strategies are not always effective and may not significantly improve the prognosis of OSCC patients [4]. While advancements in molecular research have provided valuable insights into the underlying mechanisms of this heterogeneous disease, it is still challenging to effectively apply this knowledge to improve clinical outcomes for OSCC patients.

SEC61G, also known as SEC61γ, is indeed a subunit of the SEC61 complex, along with SEC61α and SEC61β. This complex is located on the membrane of the endoplasmic reticulum (ER) and forms a translocation pore, also referred to as a hole in the middle [5]. This complex is thought to be engaged in the transportation of ER proteins, thus being regarded as an important transmembrane protein in mammals. Published studies indicated that protein transportation across the ER membranes plays a dominant role in the rapid growth of cancerous cells, and ER-associated degradation (ERAD) is mediated by proteins such as the SEC61 channel, which may be one of the potential pathways for carcinogenesis [6,7]. For example, in a study focused on lung adenocarcinoma (LUAD), SEC61G displayed the potential to enhance the abilities of proliferation, metastasis, and invasion of LUAD tumor cells via the EGFR axis [8]. Another study reported that SEC61G manipulates the proliferation and metastasis of breast cancer cells by affecting the epithelial–mesenchymal transition (EMT) process [9]. These findings indicated that SEC61G may have the potential to promote the progression of cancers. Although the SEC family has been regarded as a prognostic marker in many cancers, most studies selected to focus on SEC62 merely. The potential association between SEC61G expression levels and patient survival has not been fully investigated yet [10]. Some published studies have reported that SEC61G may serve as a prognostic indicator in different types of cancer, including glioblastoma (GBM), hepatocellular carcinoma (HCC), and kidney cancer. These data suggest that the expression level or certain characteristics of SEC61G can provide valuable information about the clinical outcomes of patients with these cancers [11,12,13]. To date, the association of SEC61G expression with OSCC has not yet been fully understood.

A greater understanding of the role of the host immune system in cancer progression has uncovered the mechanisms through which tumor cells are able to evade immune surveillance. This knowledge has laid the foundation for the discovery and advancement of targeted immunotherapies, which have demonstrated promising potential in the treatment of cancer. While PD-1 and PD-L1 targeted monoclonal antibody therapies have shown significant efficacy in some cases, it is important to note that not all patients respond favorably to this type of treatment. For instance, only a subset of patients, approximately 20% to 25%, with lung cancer exhibit a durable response to immune checkpoint inhibitors (ICIs) [14]. Indeed, the complexity of the tumor microenvironment is thought to be a key factor contributing to the variable response rates observed with immune checkpoint inhibitors and, thus, the unsatisfactory outcomes in some patients [15]. Hence, there is a pressing need to identify and explore new targets to broaden the range of effective immunotherapies. Interestingly, a recent study revealed that SEC61G can play a role in helping EGFR-amplified glioblastoma (GBM) to evade host immune elimination. The depletion of SEC61G has been found to have beneficial effects in inhibiting the occurrence of GBM. This is achieved by promoting the infiltration and cytolytic activity of CD8^+^ T cells [16]. This finding highlighted that SEC61G probably has potential immunotherapeutic value in cancer treatment. Evidence of hampered immunogenicity and heightened immune dysfunction in OSCC has indeed been observed [3]. However, the impact of SEC61G on the patients’ immune status and tumor microenvironment (TME) of OSCC remains unknown.

This study aimed to analyze the expression level of SEC61G in OSCC using data from The Cancer Genome Atlas Program (TCGA) as well as immunohistochemistry (IHC) on human OSCC tissue microarrays (TMA). In addition, the association between the expression level of SEC61G and the OSCC patients’ clinicopathological characteristics, including tumor size, lymph node involvement, clinical stage, and overall survival (OS) outcomes, were discussed. Furthermore, our study revealed a significant association between SEC61G and the decreased infiltrations of CD4^+^ and CD8^+^ lymphocytes as well as natural killer (NK) cells in human OSCC samples. Based on our research findings, it is possible to suggest that SEC61G has the potential to serve as both a prognostic biomarker and a therapeutic target for OSCC.

## 2. Material and Methods

### 2.1. Data Source

As the gene expression data and corresponding clinical information of patients were available, we collected a total of 545 gene expression samples, out of which 527 had complete clinicopathologic characteristics from the TCGA-HNSC (Head and Neck Cancer) database. The downloaded gene expression data included 501 tumor cases and 44 normal cases. Samples with incomplete gene expression data or survival information were excluded before this analysis. Data extraction was conducted using Perl (v5.30). The clinicopathologic characteristics of patients with HNSCC from the TCGA database are shown in Table 1. For immune infiltration analysis, the raw data were obtained from the TCGA database (https://portal.gdc.cancer.gov/ accessed on 4 September 2023). The scores of immune cell type in pan-caner samples from TCGA were acquired via Single-Sample Gene Set Enrichment Analysis (ssGSEA) and were further analyzed for relevance to SEC61G expression. The data of correlation between SEC61G expression and CD8A expression was from Gene Expression Profiling Interactive Analysis (GEPIA, http://gepia.cancer-pku.cn/ accessed on 4 September 2023).

### 2.2. Gene Set Enrichment Analysis (GSEA) of SEC61G

According to the expression level of *SEC61G*, we designated two sets of genes as high- and low-SEC61.

GSEA was used to compare two groups and identify significant pathways associated with *SEC61G* in HNSCC. Gene set permutations were performed 1000 times for each analysis. The nominal *p*-value, false discovery rate (FDR), and normalized enrichment score (NES) were analyzed to sort the significant pathways.

### 2.3. Ethical Statement, Human OSCC Tissue Microarray, and OSCC Patient Cohort

This study, which was performed in accordance with institutional guidelines, was approved by the Medical Ethics Committee of School and Hospital of Stomatology, Wuhan University. The approval code is 2016LUNSHENZI55, and the approval date was 26 February 2016. The human OSCC tissue microarray employed in this study was described in our previous study [17]. It consisted of 26 samples of normal oral mucosal epithelium, 21 samples of oral epithelium dysplasia, as well as 76 samples of OSCC tissues. Out of the 76 OSCC patients, 60 patients were followed up until the end of the study or death, while 16 patients were lost to follow-up. The clinicopathological parameters of the 60 primary OSCC with follow-up are shown in Table 2. The human OSCC tissues used for constructing the tissue microarray in this study were obtained from surgical specimens of OSCC patients treated at the Department of Oral Maxillofacial Head Neck Oncology, Stomatology of Wuhan University. These patients were diagnosed with OSCC by senior pathologists. Additionally, another 16 OSCC patients were enrolled in this study for immune infiltration analysis. The information and clinicopathological characteristics are displayed in Table 3. The whole blood samples were obtained before the surgery. The corresponding OSCC cancerous tissues were obtained for the following SEC61G immunohistochemical staining analysis and tumor-infiltrated lymphocyte (TIL) isolation. Informed consent was obtained from all patients.

### 2.4. Immunohistochemistry

Immunohistochemistry (IHC) was performed by using formalin-fixed, paraffin-embedded 4 µm OSCC sections. SEC61G was stained by using a rabbit anti-SEC61G polyclonal antibody (Proteintech Group Inc., Rosemont, IL, USA, 11147-2-AP), and CD8 was detected by using a mouse anti-CD8 monoclonal antibody (Maixin Biotechnology Development Co. Ltd. Fuzhou, China, MAB-1031) in this study. Briefly, the paraffin slices were first dewaxed in xylene and then dehydrated in gradient alcohol (100%, 95%, 80%, 70%) for 2 min each. The tissue sections were then rinsed with distilled water twice, each rinse lasting 5 min. Following the distilled water rinses, the tissues were rinsed with phosphate-buffered saline (PBS) three times, again for 5 min each. A pressure cooker was used for antigen retrieval with ethylenediaminetetraacetic acid (EDTA) buffer at pH 9.0 (Maixin Biotechnology Development Co. Ltd. Fuzhou, China) for a duration of 30 min. Sections were washed with PBS and incubated with the SEC61G antibody (1:100) overnight at 4 °C. On day two, sections were incubated with the enzyme-labeled anti-mouse/rabbit lgG polymer (Maixin Biotechnology Development Co. Ltd. Fuzhou, China) for 30 min at 37 °C. After washing with PBS three times, with each rinse lasting 5 min, the sections were incubated with DAB chromogenic reagent (Maixin Biotechnology Development Co., Ltd. Fuzhou, China) for an appropriate time. After the reaction with DAB, the sections were washed using PBS three times. After counterstaining with Mayer’s hematoxylin, sections were dehydrated and mounted.

### 2.5. Evaluation of Immunohistochemistry

Slides were digitally scanned using the Panoramic P250 scanner, manufactured by 3D HISTECH in Hungary. The analysis was conducted using the 3D Histech Quant Center software (Version 2.1, Budapest, Hungary). The digital tissue scanner and imaging system were used to capture scan files and images of the immunohistochemical sections. The Servicebio Image analysis system automatically measured the tissue measurement area. Expression score = ∑(pi × i) = (percentage of weak intensity area ×1) + (percentage of moderate intensity area × 2) + (percentage of strong intensity area × 3), where pi represents the percentage of the pixel area of positive signals and i represents the positive rating [18]. The expression score is a value ranging from 0 to 300, where a higher score indicates a stronger comprehensive positive intensity [19,20].

### 2.6. Peripheral Blood Mononuclear Cells (PBMCs) Separation

Firstly, whole blood samples were obtained from preoperative OSCC patients. Then, the blood samples were mixed with an equal volume of PBS with 2% fetal bovine serum (FBS) laid on the Lymphoprep^TM^ (STEMCELL Technologies, Vancouver, BC, Canada; 07801). After being centrifuged at 800× *g* for 20 min at room temperature, the layer containing PBMCs was retained for further study.

### 2.7. Tumor Infiltrated Lymphocytes (TILs) Isolation

Fresh OSCC patient-derived tumor tissues were harvested and manually cut into 2 × 1 mm pieces. Subsequently, the tissue pieces were dissociated into homogenates with a gentleMACS Dissociator (Miltenyi Biotec, Bergisch Gladbach, Germany; 130-093-235). Then, the samples were digested in RPMI medium containing Collagenase D (Roche, Basel, Switzerland; 11088858001) at 1 mg/mL, hyaluronidase at 0.1 mg/mL (Sigma–Aldrich, St. Louis, MO, USA; H1136), and DNases at 0.2 mg/mL (Sigma–Aldrich, St. Louis, MO, USA; AMPD1) for 2 h at 37 °C. After filtering the cells through 70 μm cell strainers, the obtained cells were subsequently separated using Lymphoprep^TM^ (STEMCELL Technologies, Vancouver, BC, Canada; 07801). The TILs were collected and then subjected to flow cytometry analysis.

### 2.8. Flow Cytometry

The single-cell suspension of peripheral blood mononuclear cells (PBMCs) and tumor-infiltrating lymphocytes (TILs) from patients was subjected to centrifugation and then resuspended in a staining buffer. This staining buffer contained antibodies that target cell membrane markers. The cell suspension was then incubated at a temperature of 4 °C for a duration of 30 min. Flow cytometry analysis was conducted using the CytoFLEX LX Flow Cytometer (Beckman Coulter, Brea, CA, USA). The acquired data were analyzed using the CytoExpert V10 software (Beckman Coulter, Brea, CA, USA) and the FlowJo V10 software (FlowJo™, Ashland, OR, USA). To discriminate dead cells, Fixable Viability Dye-eFluor 506 (eBioscience, San Diego, CA, USA, #65-0866-14) was used for staining. The list of antibodies is shown in Supplementary Table S2.

### 2.9. Statistical Analysis

The differences among groups of more than two were analyzed via one-way ANOVA followed by the post-Tukey test. Paired *t*-test or unpaired *t*-test was used to evaluate the difference between the two groups. The correlation analysis was performed by using two-tailed Pearson’s statistics. To analyze the overall survivals (OS) of OSCC patients, the Kaplan–Meier curves were generated and grouped using the clinical information. The cut-off is the median of the values. The receiver operating characteristic (ROC) curve was used to evaluate the predictive value of SEC61G expression. The statistical significance was identified as *p* < 0.05. All analyses were performed by using GraphPad Prism 9.0 (Graph Pad Software Inc., San Diego, CA, USA).

## 3. Results

### 3.1. The Expression Level of SEC61G Is Significantly Increased and Correlated with Clinicopathological Characteristics in Human OSCC Samples

**Figure 1:**
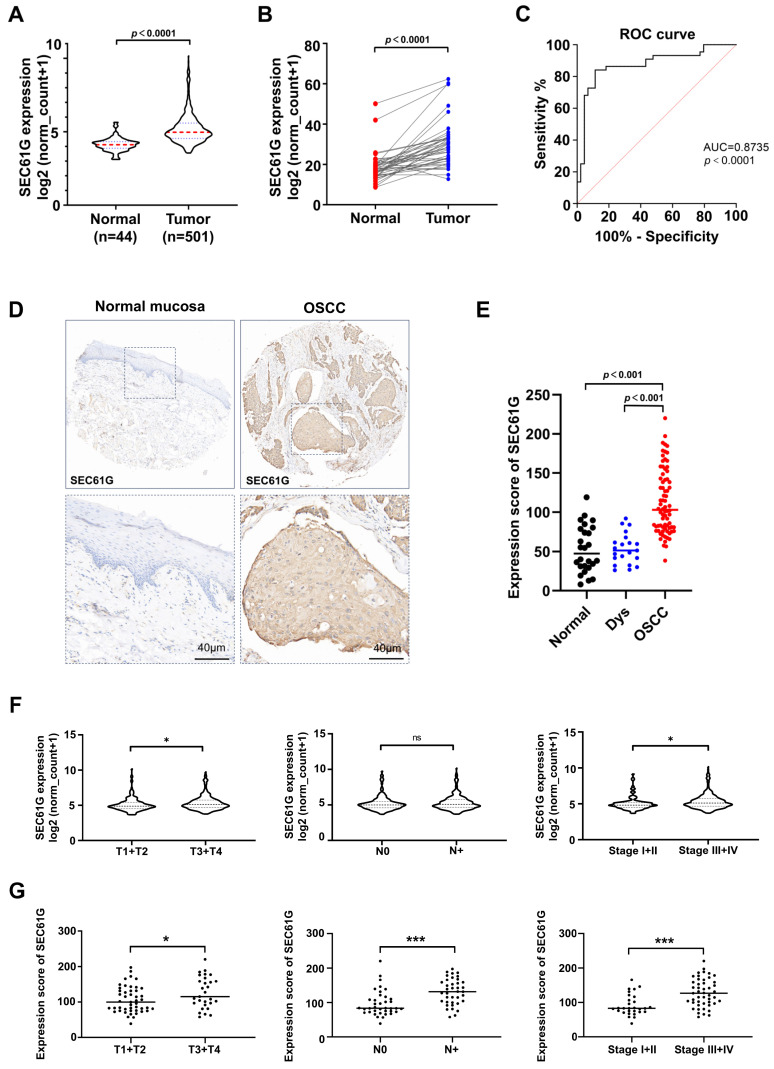
The expression level of SEC61G is significantly increased and correlates with clinicopathological characteristics in human OSCC samples. (**A**) *SEC61G* showed significantly higher expression in cancer tissues than in normal tissues (*p* < 0.0001, data from TCGA. Red dotted line means the median of the expression score, black dotted line means the Standard deviation, SD). (**B**) Comparison of *SEC61G* expression level in 40 pairs of cancer tissues and adjacent normal tissues (*p* < 0.0001, data from TCGA database). (**C**) ROC curve for *SEC61G* expression in normal tissues and cancer tissues (AUC = 0.8735, *p* < 0.0001, data from TCGA). (**D**) representative images of SEC61G IHC staining in normal oral mucosa and OSCC (scale bar = 40 µm). (**E**) The expression level of SEC61G is statistically increased in oral squamous cell carcinoma (OSCC) compared to normal oral mucosa and oral epithelial dysplasia (OSCC vs. Normal, *p* < 0.0001, OSCC vs. Dys, *p* < 0.0001). (**F**) Data from the TCGA database indicated that *SEC61G* expression level is significantly associated with tumor size (*p* < 0.05, T1 + T2 vs. T3 + T4) and clinical stage (*, *p* < 0.05, Stage I + II vs. Stage III + IV), but not lymph node involvement (N0 vs. N+, ns, no significance). (**G**) IHC analysis indicated that SEC61G expression level is significantly associated with tumor size (*, *p* < 0.05, T1 + T2 vs. T3 + T4), lymph node involvement (***, *p* < 0.001, N0 vs. N+), and clinical stage (***, *p* < 0.001, Stage I + II vs. Stage III + IV).

A total of 545 *SEC61G* expression data from TCGA were analyzed in this study, including 501 in HNSCC and 44 in normal tissues. As shown in Figure 1A, the result indicated that higher expression of *SEC61G* was correlated significantly with the tumor group (*p* < 0.0001). To obtain a more reliable conclusion, we compared *SEC61G* expression in tumor tissue and adjacent normal tissue from the same patients using a paired *t*-test. It also demonstrated that *SEC61G* expression was higher in tumor tissues compared with their normal control (Figure 1B, *p* < 0.0001). Then, receiver operating characteristic (ROC) curves were utilized to evaluate the diagnostic value of *SEC61G*. We discovered that the area under the curve (AUC) was 0.87 (Figure 1C, *p* < 0.0001), indicating that *SEC61G* exhibited significant diagnostic value. To validate the results obtained from TCGA, we conducted immunohistochemistry (IHC) analysis on our human OSCC tissue microarray (TMA). The representative images of normal mucosa and OSCC tissue are displayed in Figure 1D. SEC61G expression was predominantly observed in the epithelial region of cancerous tissues and primarily localized in the cytoplasm of tumor cells. Statistically, there was a notable increase in SEC61G expression in OSCC tissues when compared to oral epithelium dysplasia (Dys) and normal mucosa (Figure 1E, *p* < 0.001). This observation suggests that SEC61G may play a potential pro-oncogenic role in OSCC. Additionally, the correlation between the expression level of SEC61G and the clinicopathological characteristics of OSCC patients was analyzed. Data from TCGA showed a significant correlation between *SEC61G* expression and tumor size (T1 + T2 vs. T3 + T4) as well as clinical stage (Stage I + II vs. Stage III + IV) in OSCC patients (Figure 1F, *p* < 0.05). However, there was no statistically significant correlation between *SEC61G* expression and lymph node involvement (N0 vs. N+, Figure 1F). Of note, there was a partial inconsistency between the data from TCGA and our TMA results. Our TMA analysis revealed that SEC61G was significantly correlated with tumor size (Figure 1G, right, *p* < 0.05, T1 + T2 vs. T3 + T4), lymph node involvement (Figure 1G, middle, *p* < 0.001, N0 vs. N+), and clinical stage (Figure 1G, left, *p* < 0.001, Stage I + II vs. Stage III + IV). These findings indicate a significant increase in SEC61G expression and suggest its potential involvement in the progression of OSCC.

Additionally, by using the TCGA-HNSC database, we performed GSEA and illustrated significant pathways in the enrichment of MSigDB Collections (c2.cp.kegg.v7.1.symbols.gmt and h.all.v7.0.symbols.gmt). FDR q-value < 0.05 and NOM *p*-value < 0.05 were used for screening, and 11 pathways showed significantly differential enrichment in the *SEC61G* high-expression group (Supplementary Figure S1 and Table S1). Several tumor-related biological pathways were listed, such as oxidative phosphorylation, myc target, and DNA repair, indicating the potential role of *SEC61G* in the development of OSCC patients.

### 3.2. Prognostic Value of SEC61G in OSCC

**Figure 2:**
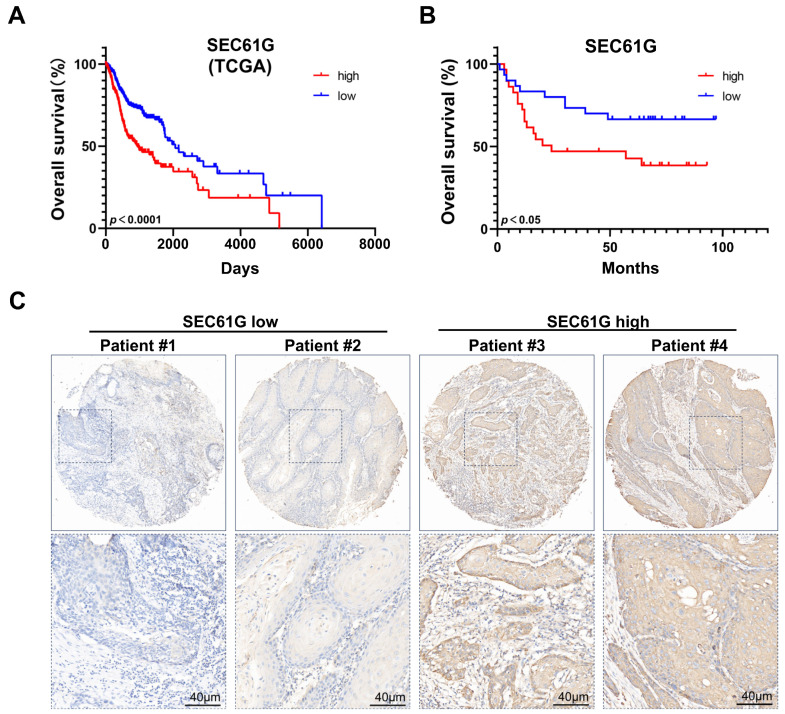
Prognostic value of SEC61G in OSCC. (**A**) Kaplan–Meier curves showed the impact of *SEC61G* expression on overall survival (OS, data from TCGA, *p* < 0.0001, cut-off is the median of SEC61G expression value). (**B**) Impact of SEC61G expression on OS (data from IHC analysis, *p* < 0.05, cut-off is the median of SEC61G expression score). (**C**) Representative IHC images of SEC61G low expression (right) and high expression (left, scale bar = 40 µm).

Initially, we utilized the TCGA-HNSC database to examine the prognostic significance of *SEC61G* expression. Kaplan–Meier curves revealed that the patients exhibiting high expression levels of *SEC61G* had poorer overall survival (OS) compared to those with low *SEC61G* expression (Figure 2A, *p* < 0.0001). Subsequently, we employed the median expression score as a cut-off to assess the influence of SEC61G on prognosis within our patient cohort. Consistent with the findings from the TCGA database, we observed a significant association between higher SEC61G expression levels and worse outcomes in our patient cohort (Figure 2B, *p* < 0.05). Figure 2C displays representative immunohistochemistry (IHC) images showcasing high and low SEC61G expression levels. Furthermore, through a more comprehensive analysis of the TCGA database, we performed subgroup analyses considering important clinicopathological characteristics in order to gain insights into specific patient groups. In these results, high *SEC61G* expression significantly affected the OS in the patients of T1/T2 (*p* < 0.01), T3/T4 (*p* < 0.05), N0 (*p* < 0.05), N1+ (*p* < 0.01), M0 (*p* < 0.05), and Stage III/IV (*p* < 0.001) (Supplementary Figure S2A–D). Additionally, our findings solidify the notion that *SEC61G* expression serves as a prognostic indicator in patients who did not undergo radiotherapy (Supplementary Figure S2E, *p* < 0.0001). However, in the group of patients who received radiotherapy, *SEC61G* expression did not demonstrate significant prognostic value (Supplementary Figure S2F). Based on the aforementioned findings, it can be concluded that SEC61G can be considered a prognostic biomarker in patients with OSCC.

### 3.3. SEC61G Expression Level Is Negatively Associated with Immunostimulation and Immune Cell Infiltration in OSCC

**Figure 3:**
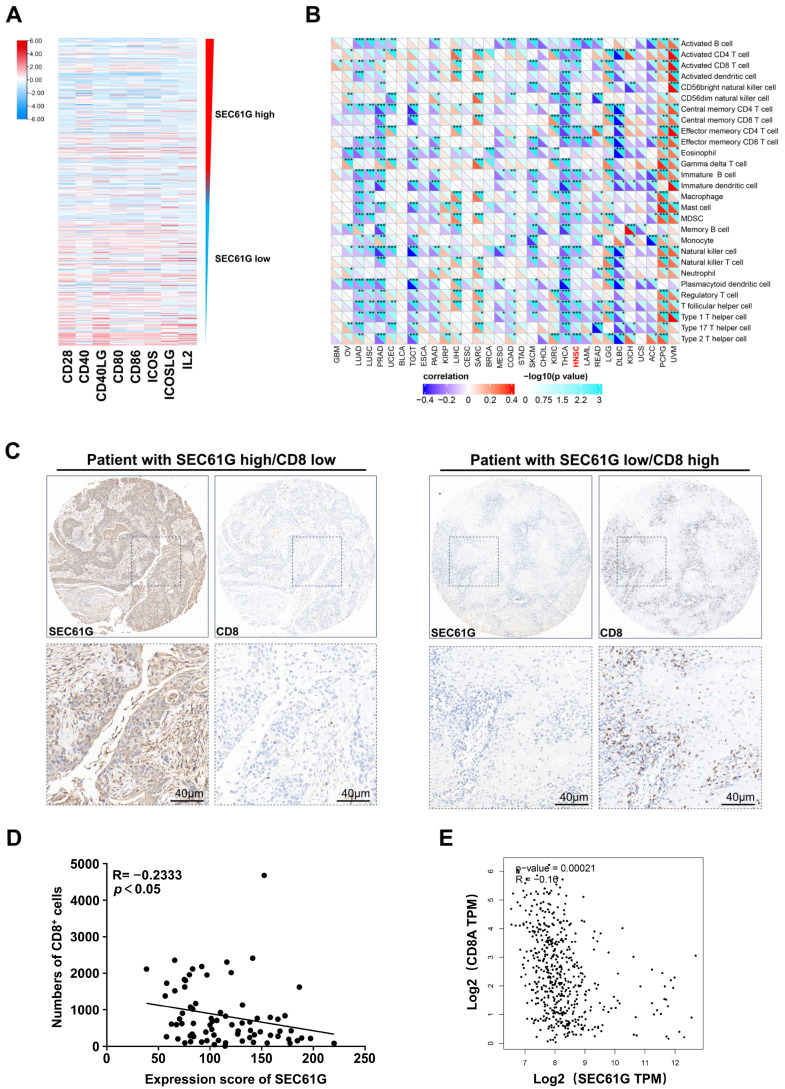
SEC61G expression level is negatively associated with immunostimulation and immune cell infiltration in OSCC. (**A**) Expression of genes related to immunostimulation in different classifications based on *SEC61G* expression. (**B**) The correlations between *SEC61G* expression and immune cell scores based on ssGSEA in pan-cancer samples from TCGA, (*, *p* < 0.05, **, *p* < 0.01, ***, *p* < 0.001). (**C**) Representative images of serial sections IHC images of SEC61G and CD8 (scale bar = 40 µm). (D) SEC61G expression is negatively correlated with the number of CD8 positive cells in human OSCC samples (*p* < 0.05, r = −0.2333). (**E**) Data on the correlation between SEC61G and CD8A are from GEPIA (*p* = 0.00021, r = −0.16. http://gepia.cancer-pku.cn/ accessed on 4 September 2023).

Due to its strong association with immune evasion in glioblastoma, we used bioinformatics databases to investigate the relationship between SEC61G and immunostimulation as well as immune cell infiltrations. The analysis showed a negative correlation between *SEC61G* expression and immunostimulation-related genes in the HNSCC sample from the TCGA database. The single-sample gene set enrichment analysis (ssGSEA) further demonstrated that high expression of *SEC61G* was associated with a decrease in the infiltration of activated CD8^+^ T cells, activated CD4^+^ T cells, NK cells, activated B cells, and so on. These findings suggest that overexpression of *SEC61G* is indicative of an immunosuppressive state. The subsequent immunohistochemistry (IHC) analysis performed on the serial sections revealed a statistically significant correlation between SEC61G expression level and a reduction in the number of CD8^+^ cells (Figure 3D, *p* < 0.05, r = −0.2333, two-tailed Pearson’s statistics). The representative images of serial sections IHC images are displayed in Figure 3C. We also checked the GEPIA database and found *SEC61G* mRNA expression was negatively related to CD8A mRNA expression (Figure 3E, *p* = 0.00021, r = −0.16).

### 3.4. SEC61G Shows a Negative Correlation with the Accumulation of Immune Cells in OSCC Patients’ Samples

**Figure 4:**
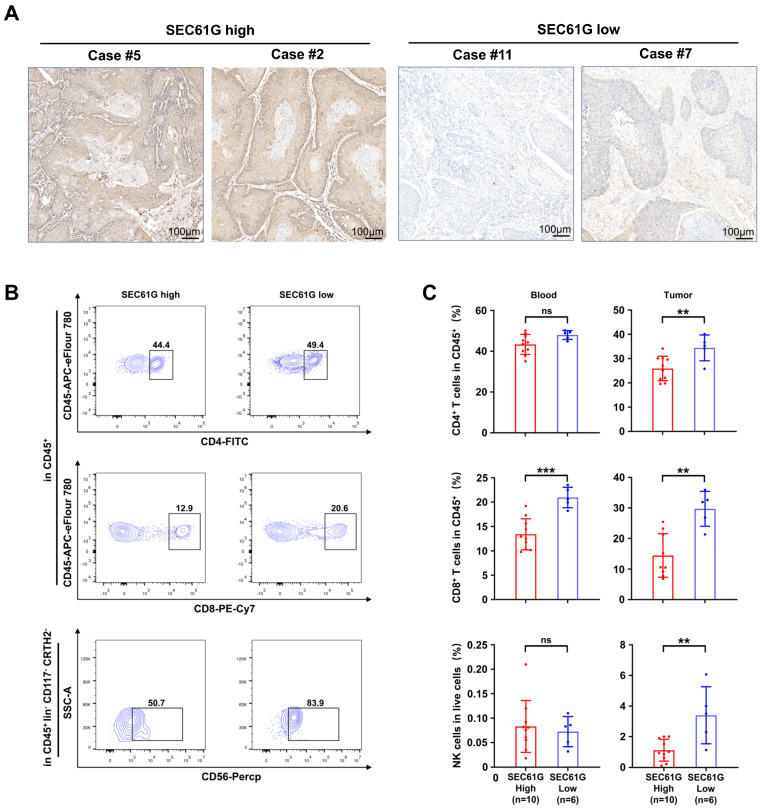
SEC61G shows a negative correlation with the accumulation of immune cells in OSCC patients’ samples. (**A**) Representative IHC images of SEC61G high expression (right) and SEC61G low expression (left, scale bar = 100 µm). (**B**) Representative flow cytometry images of CD4^+^ T cells, CD8^+^ T cells, and NK cells. (**C**) Quantification of the frequency of CD4^+^ T cells, CD8^+^ T cells, and NK cells in OSCC patient-derived blood and tumor-infiltrated lymphocytes (TILs, **, *p* < 0.01, ***, *p* < 0.001, ns, no significance).

To investigate the influence of SEC61G expression on immune cell infiltration, we collected preoperative peripheral blood and paired postoperative cancerous tissues from 16 patients with OSCC. The OSCC cancerous tissue was divided into two parts for separate analysis: one part for IHC analysis and the other part for TIL isolation. IHC analysis of these 16 OSCC samples was carried out using the same methods as the tissue microarray (TMA) analysis. The median of the SEC61G expression score was used as the cut-off to classify patients into two groups: SEC61G high (*n* = 10) and SEC61G low (*n* = 6). The representative images of SEC61G high and SEC61G low are displayed in Figure 4A. Subsequently, PBMCs and TILs were subjected to flow cytometry analysis. Two T cell phenotypes were identified by using CD4 and CD8 staining. NK cells were defined as CD45^+^CD3^-^CD19^-^CD5^-^CD34^-^CRTH2^-^CD117^-^CD56^+^ cells in PBMCs and TILs. Representative images of cytometric analysis for CD4^+^ T cells, CD8^+^ T cells, and NK cells are displayed in Figure 4B. The gating strategies for CD4^+^, CD8^+^, and NK cells are presented in Supplementary Figure S3. Our findings demonstrate a significant correlation between high SEC61G expression and reduced infiltration of CD4^+^ T cells, CD8^+^ T cells, as well as NK cells within the TILs. It is important to note, however, that high SEC61G expression only showed an association with decreased numbers of CD8^+^ T cells within the PBMCs. These results provide evidence linking SEC61G expression with a decreased anti-tumor immune response in patients with OSCC.

## 4. Discussion

SEC61G was reported to be overexpressed in various types of cancers. Up-regulation of SEC61G in cancer suggests its potential involvement in tumor development or progression [9,10,11,13]. However, the specific role and underlying mechanisms of SEC61G in different cancer types require further investigation. Considering the expression of SEC61G in OSCC is unclear, in the present study, we attempt to assess SEC61G expression by using two approaches. Firstly, we utilized data from The Cancer Genome Atlas (TCGA), a comprehensive cancer database, to analyze SEC61G expression at the mRNA level in OSCC patients. Additionally, immunohistochemistry (IHC) was performed on OSCC tissue microarrays, allowing for the evaluation of SEC61G protein levels in a relatively large number of patient samples. By utilizing both TCGA data and IHC, we obtained a comprehensive understanding of SEC61G expression, which was significantly increased in OSCC patients. These findings indicated that up-regulation was the most common alteration of SEC61G in OSCC and provided additional evidence supporting its potential tumorigenic role in this specific cancer type. This conclusion is consistent with previous studies conducted on other cancer types, such as breast cancer, lung cancer, kidney cancer, and glioblastoma [8,9,13,21]. Notably, our study findings obtained through both bioinformatics analysis and immunohistochemistry (IHC) indicated that increased SEC61G expression levels are significantly correlated with poorer clinicopathologic factors in oral squamous cell carcinoma (OSCC). These factors include larger tumor size, lymph node metastasis, and advanced clinical stage. This correlation suggests that elevated SEC61G expression may serve as a potential pro-oncogenic marker in OSCC, indicating a more aggressive phenotype of the disease. These findings were also supported by two additional studies conducted on SEC61G in head and neck squamous cell carcinomas (HNSCC) [22,23]. Compared to these two studies, in addition to the bioinformatics research, our study conducted more comprehensive analyses of the clinical samples of patients with OSCC. Nevertheless, further validation and exploration are warranted to fully elucidate the molecular mechanisms underlying these associations.

In the present study, although there is currently a lack of systematic assays to identify the underlying molecular mechanisms of SEC61G in oral squamous cell carcinoma (OSCC), we utilized Gene Set Enrichment Analysis (GSEA) to gain further insights into its potential role in the development of OSCC. Bioinformatics data indicated SEC61G’s high expression was associated with MYC, oxidative stress, and DNA repair in OSCC. MYC is a proto-oncogene family consisting of several members, including I-MYC, N-MYC, and C-MYC [24]. Several reports have indeed confirmed the genetic alterations of the MYC gene in oral cancer. The MYC gene, specifically the c-MYC oncogene, plays a crucial role in regulating cell growth, proliferation, and apoptosis. Aberrant expression or amplification of the MYC gene has been observed in oral squamous cell carcinoma (OSCC) and is associated with tumor development and progression [25]. Oxidative stress refers to an imbalance between the production of reactive oxygen species (ROS) and the body’s ability to detoxify or repair the resulting damage. Excessive ROS can lead to cellular damage, DNA mutations, and alterations in cellular signaling pathways, thereby promoting cancer development and progression [26]. Additionally, it has been suggested that the DNA repair defective cell lines are more active and invasive in vitro, and the poor prognosis of HNSCC is obviously associated with DNA-repair-related gene defects [27]. Hence, exploring the relationship among SEC61G, the MYC gene, oxidative stress, and DNA repair in future studies would be valuable.

Investigating novel prognostic markers in cancer indeed provides crucial information about the aggressiveness of the disease and the expected clinical outcome of individual patients, especially in the absence of treatment. Prognostic markers help in stratifying patients into different risk groups, which can guide treatment decisions and improve patient care. Despite significant advancements in the treatment strategies for OSCC over the past few decades, clinical outcomes still leave room for improvement [4]. This might be associated with the heterogeneity observed in tumors. Previous studies have indeed suggested that SEC61G can serve as an effective marker for predicting worse prognosis in several cancer types, including lung cancer, breast cancer, kidney cancer, head and neck cancer, and hepatocellular carcinoma (HCC). This is due to the role of SEC61G in promoting cancer cell proliferation, migration, and the epithelial–mesenchymal transition (EMT) process [8,9,12,13]. EMT is an important mechanism in normal embryonic development and tissue regeneration [28]. However, the abnormal reactivation of the EMT during cancer progression is firmly linked to the acquisition of malignant characteristics by tumor cells [29]. In the present study, while the results consistently support the notion that SEC61G expression is a predictor of clinical outcome in patients with oral squamous cell carcinoma (OSCC), it is important to highlight that there is still a lack of investigation regarding the bio-functional role of SEC61G in OSCC cells. The relationship between SEC61G and oncogenic mechanisms, particularly in the context of EMT, is of great importance and warrants further exploration.

The advent of immunotherapy has indeed brought increased attention to the tumor microenvironment. Studies have consistently demonstrated that immune cells constitute a significant portion of the tumor microenvironment (TME) and play a crucial role in tumor development [30]. The TME is a complex ecosystem composed of various cell types, including immune cells, stromal cells, fibroblasts, and blood vessels [31]. Among others, immune cells have garnered significant interest due to their ability to interact with tumor cells and influence tumor behavior. The abundance of CD8^+^ T cells in the tumor microenvironment (TME) has consistently been regarded as a positive prognostic marker for various types of cancer [32]. A recent study investigating glioblastoma has provided evidence for the negative modulatory function of SEC61G. Deletion of SEC61G in mouse glioblastoma induced increased infiltration of CD8^+^ cytotoxic T cells in TME [16]. Furthermore, bioinformatics studies on HNSCC have also indicated a potential correlation between the expression level of SEC61G and the infiltration of immune cells [22,23]. In the present study, an analysis of data from The Cancer Genome Atlas (TCGA) revealed a negative correlation between SEC61G and several co-stimulatory pathways, including CD28, CD40, CD80, CD86, ICOS, and IL-2 [33]. Furthermore, SEC61G was also found to be negatively associated with the infiltration of immune cells such as activated CD8^+^ T cells, activated CD4^+^ T cells, and NK cells. These findings were partially consistent with previous studies, suggesting that SEC61G may play a role in modulating the immune response within the TME. The negative correlation with co-stimulatory pathways implies that higher levels of SEC61G may be associated with reduced immune activation, potentially leading to a less favorable anti-tumor immune response. Notably, OSCC is a type of cancer that is characterized by impaired host immunity and a heterogeneous TME [3]. Growing evidence has demonstrated a significant association between the presence of TILs and prognosis in various solid tumors, including OSCC [34]. CD8^+^ lymphocytes have been consistently associated with a favorable prognosis in OSCC [35,36]. Through IHC analysis on our OSCC tissue microarrays (TMAs), we found that SEC61G expression levels are negatively correlated with the numbers of CD8^+^ cells. This finding implies that SEC61G may have a negative immunomodulatory effect in OSCC. SEC61G expression may influence the recruitment or activation of CD8^+^ cells. Further flow cytometry assays conducted on PBMCs and ITLs from OSCC patients have provided more robust validation for the conclusion. The negative correlation between SEC61G expression levels and the infiltration of CD4^+^ T cells, CD8^+^ cells, and NK cells in OSCC patients indicates that SEC61G may be involved in immune evasion mechanisms employed by the tumor. Our data strongly suggest a potential immunotherapeutic role for SEC61G in OSCC. While our current data provide valuable insights, more extensive studies are required to fully understand the underlying mechanisms and assess the therapeutic efficacy and safety of targeting SEC61G.

While we conducted thorough analyses of SEC61G expression using data from clinical samples and public databases, it is important to note that our study has certain limitations. Further research is needed to delve into the functions and molecular mechanisms of SEC61G in OSCC. In addition, it is necessary to conduct in vivo experimental therapies to assess the immunotherapeutic potential of targeting SEC61G or combining it with anti-PD-1 treatment. These experiments will allow us to evaluate the efficacy and safety of such therapeutic strategies and provide insights into their potential clinical applications. This additional investigation will provide a more comprehensive understanding of the role of SEC61G in OSCC and its potential implications for diagnosis, prognosis, and targeted therapies.

## 5. Conclusions

In our study, we employed bioinformatics analysis and immunohistochemistry (IHC) to estimate the expression level of SEC61G in OSCC patients. The findings of our study demonstrated a significant amplification of SEC61G expression in OSCC. Furthermore, we observed a correlation between SEC61G expression levels and the clinicopathological characteristics of OSCC patients, as well as their prognoses. Additionally, our study revealed a negative correlation between SEC61G expression and the presence of CD4^+^ T cells, CD8^+^ T cells, and NK cells in OSCC human samples. This finding suggests a potential immunosuppressive role of SEC61G in the tumor microenvironment of OSCC. Despite the presence of some limitations, our study provided compelling evidence supporting the role of SEC61G as a diagnostic, prognostic, and therapeutic biomarker in OSCC.

## Figures and Tables

**Table 1:** Clinicopathologic characteristics of patients with HNSCC from TCGA.

Characteristics		Cases	Percentages (%)
Age	<65 years	330	62.62%
	≥65 years	196	37.19%
	Unknown	1	0.19%
Gender	Male	385	73.06%
	Female	142	26.94%
Histological grade	G1	63	11.95%
	G2	311	59.01%
	G3	124	23.53%
	G4	7	1.33%
	GX	18	3.42%
	Unknown	4	0.76%
Clinical stage	I/II	101	19.17%
	III/IV	351	66.60%
	Unknown	75	14.23%
Tumor size	T0	1	0.19%
	T1/T2	189	35.86%
	T3/T4	275	52.18%
	TX	39	7.40%
	Unknown	23	4.36%
Lymphoid nodal status	N0	179	33.97%
	N1+	248	47.06%
	NX	75	14.23%
	Unknown	25	4.74%
Distant metastasis status	M0	190	36.05%
	M1	1	0.19%
	MX	65	12.33%
	Unknown	271	51.42%
Radiation therapy	Yes	203	38.52%
	No	324	61.48%
Survival status	Death	199	37.76%
	Survival	328	62.24%

**Table 2:** Clinicopathologic characteristics of patients with OSCC in a tissue microarray (TMA).

No.	Gender	Age	TNM Stage	Grade	Alive (0) or Dead (1)	Survival Time (Months)
1	male	49	T2N0M0	I	0	74
2	male	50	T2N1M0	III	1	49
3	male	50	T3N1M0	II	1	9
4	female	43	T2N0M0	II	0	72
5	female	65	T3N0M0	II	1	13
6	female	73	T3N0M0	II	0	68
7	male	40	T3N2M0	II	1	4
8	male	38	T2N1M0	I	0	31
9	male	44	T1N0M0	II	0	59
10	female	67	T2N0M0	II	0	51
11	male	73	T2N1M0	I	0	45
12	male	61	T1N0M0	II	1	39
13	male	68	T2N0M0	II	0	39
14	female	57	T1N0M0	II	0	65
15	male	60	T3N0M0	III	1	3
16	male	40	T2N1M0	II	0	97
17	male	39	T2N0M0	II	0	96
18	male	77	T1N0M0	III	1	5
19	male	68	T2N2M0	I	0	93
20	male	63	T3N1M0	I	1	20
21	male	43	T2N0M0	III	1	30
22	female	78	T4N2M0	II	0	88
23	male	57	T3N1M0	II	1	0
24	male	72	T4N0M0	II	0	85
25	male	62	T4N1M0	II	1	24
26	male	80	T4N0M0	II	1	12
27	male	70	T4N1M0	II	0	84
28	male	72	T2N2M0	II	0	29
29	male	57	T3N1M0	II	0	83
30	male	53	T3N1M0	IIII	1	12
31	male	55	T3N0M0	II	1	8
32	female	66	T1N1M0	III	1	16
33	male	62	T1N0M0	II	0	82
34	male	46	T4N0M0	II	0	82
35	male	54	T4N1M0	I	1	11
36	male	54	T2N1M0	I	0	79
37	male	41	T2N1M0	III	0	76
38	male	46	T2N1M0	II	1	7
39	male	62	T2N0M0	III	1	4
40	male	49	T2N0M0	II	0	73
41	male	78	T2N1M0	I	0	73
42	male	63	T4N2M0	II	1	64
43	male	48	T3N0M0	II	0	70
44	female	65	T4N2M0	I	0	69
45	male	57	T4N1M0	III	1	57
46	female	58	T2N0M0	II	0	68
47	male	35	T2N0M0	II	0	68
48	male	50	T4N0M0	III	0	67
49	male	48	T2N1M0	II	0	11
50	male	57	T2N0M0	II	0	67
51	male	52	T2N0M0	III	1	21
52	male	59	T2N2M0	II	1	9
53	female	46	T2N0M0	II	1	30
54	male	51	T4N1M0	I	0	65
55	male	61	T1N0M0	II	1	1
56	male	46	T4N1M0	II	0	63
57	male	47	T2N2M0	II	1	3
58	male	63	T4N2M0	II	1	4
59	male	61	T2N0M0	III	1	10
60	male	69	T2N2M0	II	1	17

**Table 3:** Clinicopathologic characteristics of patients for immune infiltration analysis.

No.	Gender	Age	TNM Stage	Grade	Stage
1	male	50	T3N2bM0	II	IVA
2	male	39	T3N1M0	III	III
3	male	38	T1N0M0	I	I
4	male	48	T2N0M0	III	II
5	male	43	T3N1M0	II	III
6	female	57	T2N0M0	II	II
7	male	64	T1N1M0	II	III
8	male	38	T3N1M0	II	III
9	male	54	T3N0M0	II	III
10	female	74	T3N0M0	II	III
11	male	70	T2N1M0	II	III
12	male	64	T3N0M0	I	III
13	male	57	T2N3bM0	III	IVB
14	female	53	T2N1M0	II	III
15	male	38	T3N1M0	III	III
16	male	74	T2N0M0	I	II

## Data Availability

The data presented in this study are available on request from the corresponding author.

## Supplementary Materials

The following supporting information can be downloaded at https://www.mdpi.com/article/10.3390/biomedicines11102718/s1, Figure S1: Enrichment plots from gene set enrichment analysis (GSEA) of SEC61G. Figure S2: Kaplan–Meier curves of overall survival (OS) in subgroups of HNSCC patients. Figure S3. Gating strategies of immune cells. Table S1. Gene sets enriched in the high SEC61G expression. Table S2. List of antibodies for flow cytometry.
