# Supplementary material for: Identification of SEC61G as a Diagnostic and Prognostic Biomarker in Oral Squamous Cell Carcinoma
Source: Biomedicines. 2023 Oct 6;11(10):2718. doi: 10.3390/biomedicines11102718 (PMC10603851; doi:10.3390/biomedicines11102718)
Supplement: Supplementary file 1 [file biomedicines-11-02718-s001.zip › biomedicines-2622167-supplementary.pdf]

Supplementary Table S1. Gene sets enriched in the high SEC61G expression group.

Gene set name	NES	NOM *P*-value	FDR q-value
HALLMARK_OXIDATIVE_PHOSPHORYLATION	2.091	0.000	0.006
HALLMARK_MYC_TARGETS_V1	1.888	0.008	0.025
HALLMARK_MYC_TARGETS_V2	1.875	0.010	0.019
HALLMARK_DNA_REPAIR	1.859	0.006	0.017
KEGG_OXIDATIVE_PHOSPHORYLATION	2.233	0.000	0.000
KEGG_PARKINSONS_DISEASE	2.103	0.002	0.004
KEGG_PROTEASOME	2.004	0.002	0.009
KEGG_RIBOSOME	1.993	0.000	0.009
KEGG_HUNTINGTONS_DISEASE	1.970	0.006	0.009
KEGG_ALZHEIMERS_DISEASE	1.953	0.008	0.009
KEGG_PROTEIN_EXPORT	1.917	0.000	0.013

NES: normalized enrichment score; FDR: false discovery rate.

Supplementary Table S2. List of antibodies for flow cytometry.

Antibody/Fluorophore	Target Species	Company	Clone	Catalog Number
CD45-APC-eFluor 780	human	eBioscience, San Diego CA	HI30	47-0459-41
CD3-Alexa Fluor 700	human	eBioscience, San Diego CA	UCHTI	56-0038-41
CD3-FITC	human	BioLegend, San Diego CA	OKT3	317305
CD8-PE-Cy7	human	eBioscience, San Diego CA	SK1	25-0087-42
CD4-FITC	human	BD Biosciences, La Jolla CA	RPA-T4	561005
CD34-FITC	human	BioLegend, San Diego CA	561	343603
CD5-FITC	human	BioLegend, San Diego CA	L17F12	364021
CRTH2-PE-Cy7	human	BioLegend, San Diego CA	BM16	350117
CD56-PerCP	human	BioLegend, San Diego CA	5.1H11	362525
CD19-FITC	human	BioLegend, San Diego CA	H1B19	302205
CD117-BV421	human	BioLegend, San Diego CA	104D2	313215

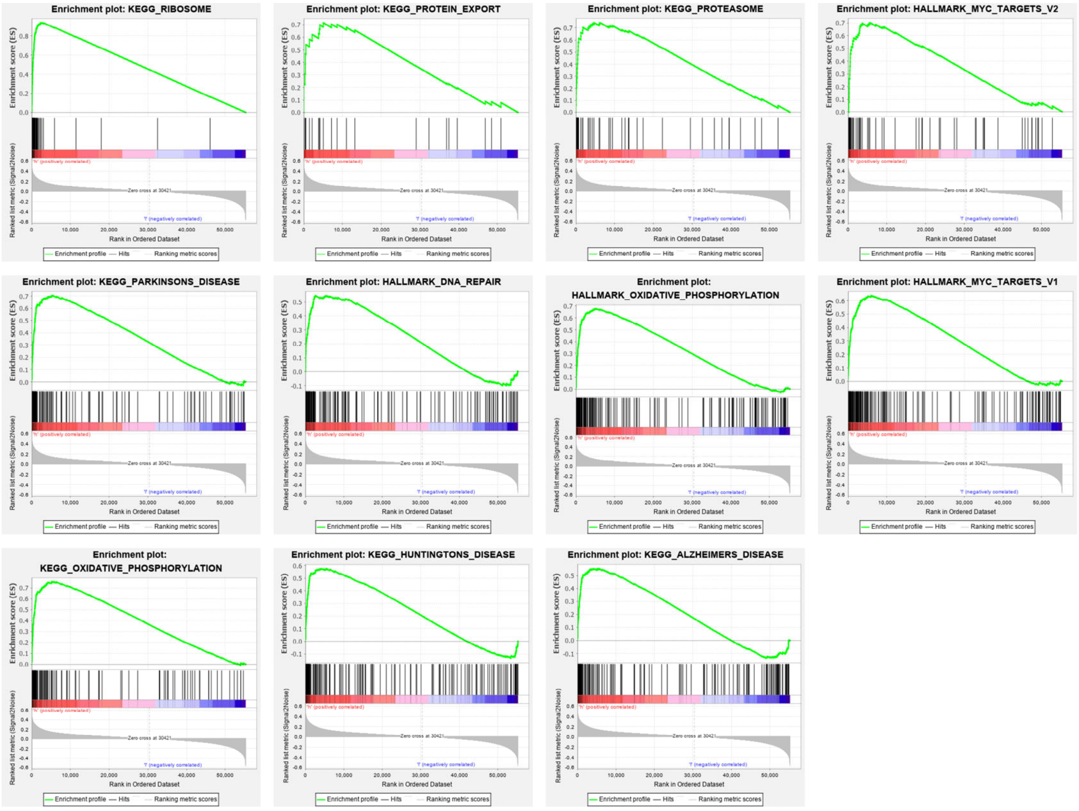

Supplementary Figure S1. Enrichment plots from gene set enrichment analysis (GSEA) of SEC61G. GSEA results showing differential enrichment of genes related to oxidative phosphorylation, MYC targets, DNA repair, Parkinson's disease, proteasome, ribosome, Huntington's disease, Alzheimer's disease, and protein export in HNSCC with high SEC61G expression.

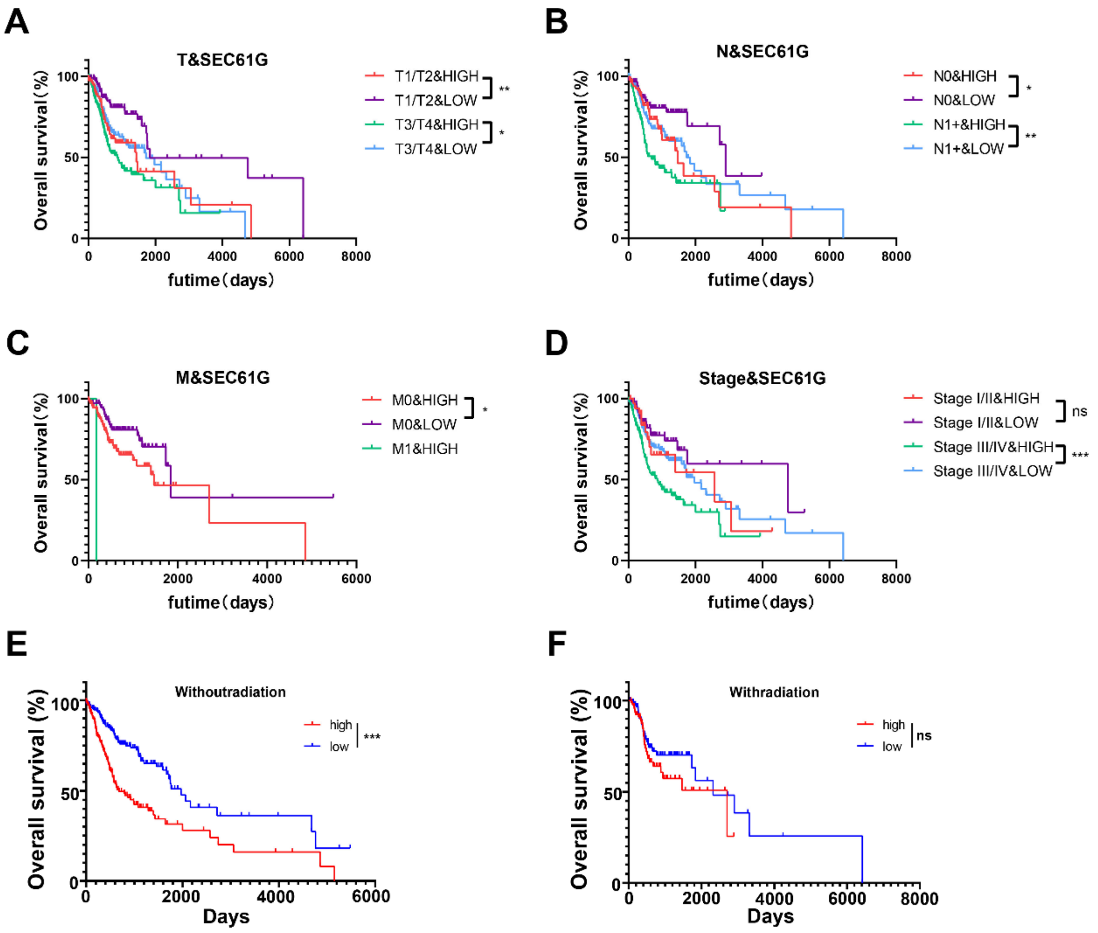

Supplementary Figure S2. Kaplan–Meier curves of overall survival (OS) in subgroups HNSCC patients. A. Impact of SEC61G expression and T stage on OS. B. Impact of SEC61G expression and N stage on OS. C. Impact of SEC61G expression and M stage on OS. D. Impact of SEC61G expression and clinical stage on OS. E. The prognostic value of SEC61G expression in patients without radiotherapy. F. The prognostic value of SEC61G expression in patients with radiotherapy. *, $P<0.05$, **, $P<0.01$, ***, $P<0.001$, ns, no significance.

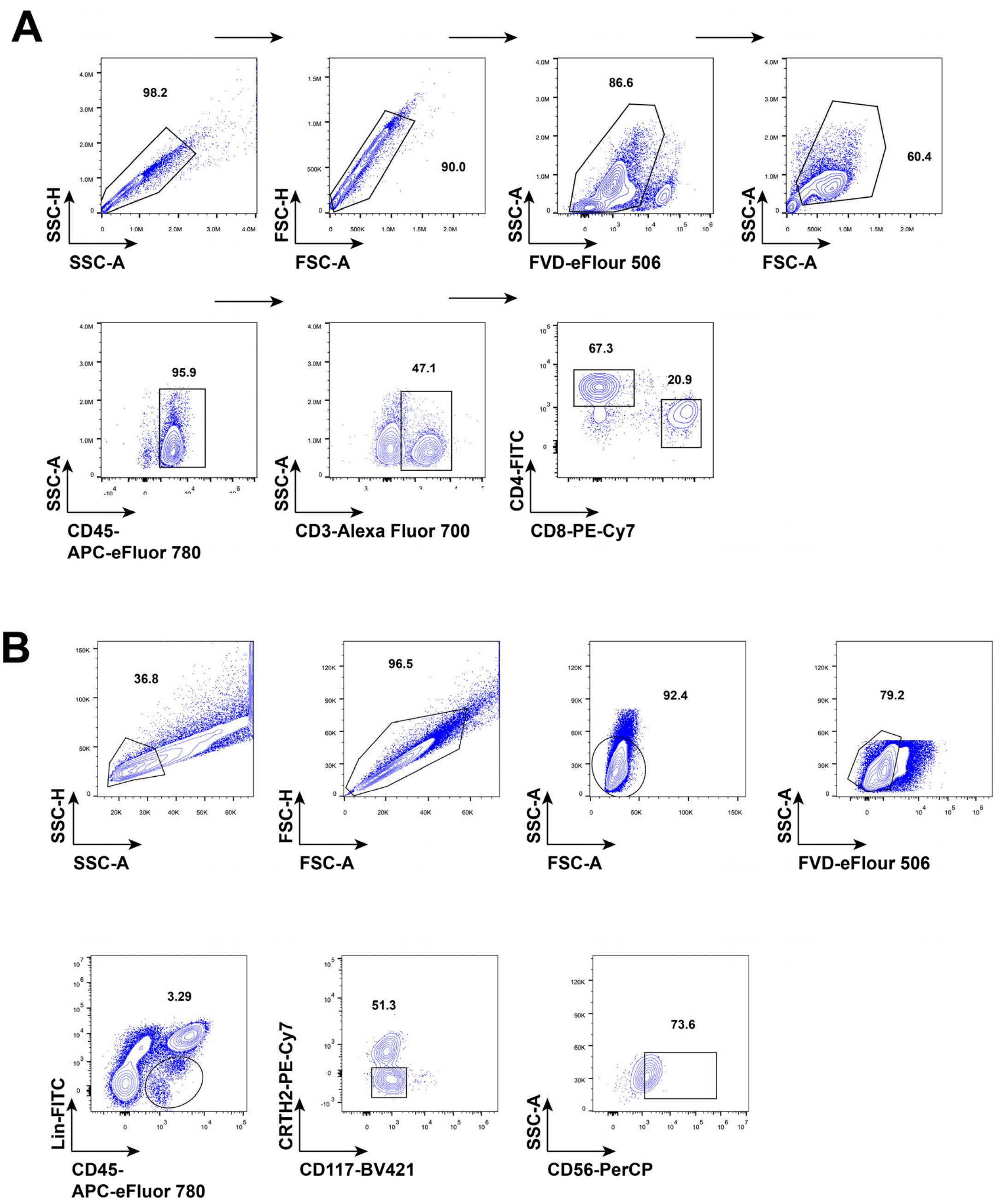

Supplementary Figure S3. Gating strategies of immune cells in human OSCC samples. A. Gating strategy of CD4⁺ and CD8⁺ T cells. B. Gating strategy of NK cells.
